# Evidence for a Resting State Network Abnormality in Adults Who Stutter

**DOI:** 10.3389/fnint.2018.00016

**Published:** 2018-04-27

**Authors:** Amir H. Ghaderi, Masoud N. Andevari, Paul F. Sowman

**Affiliations:** ^1^Cognitive Neuroscience Laboratory, University of Tabriz, Tabriz, Iran; ^2^Iranian Neuro-wave Laboratory, Center of Isfahan, Isfahan, Iran; ^3^Department of Physics, School of Basic Science, Babol Noshirvani University of Technology, Babol, Iran; ^4^Department of Cognitive Science, Faculty of Human Sciences, Macquarie University, Sydney, NSW, Australia; ^5^ARC Centre of Excellence in Cognition and its Disorders, Macquarie University, Sydney, NSW, Australia

**Keywords:** stuttering, functional brain networks, minimum spanning tree, executive function, time perception

## Abstract

Neural network-based investigations of stuttering have begun to provide a possible integrative account for the large number of brain-based anomalies associated with stuttering. Here we used resting-state EEG to investigate functional brain networks in adults who stutter (*AWS*). Participants were 19 *AWS* and 52 age-, and gender-matched normally fluent speakers. *EEGs* were recorded and connectivity matrices were generated by *LORETA* in the theta (4–8 Hz), alpha (8–12 Hz), beta1 (12–20 Hz), and beta2 (20–30 Hz) bands. Small-world propensity (*SWP*), shortest path, and clustering coefficients were computed for weighted graphs. Minimum spanning tree analysis was also performed and measures were compared by non-parametric permutation test. The results show that small-world topology was evident in the functional networks of all participants. Three graph indices (diameter, clustering coefficient, and shortest path) exhibited significant differences between groups in the theta band and one [maximum betweenness centrality (*BC*)] measure was significantly different between groups in the beta2 band. *AWS* show higher *BC* than control in right temporal and inferior frontal areas and lower *BC* in the right primary motor cortex. Abnormal functional networks during rest state suggest an anomaly of *DMN* activity in *AWS*. Furthermore, functional segregation/integration deficits in the theta network are evident in *AWS*. These deficits reinforce the hypothesis that there is a neural basis for abnormal executive function in *AWS*. Increased beta2 *BC* in the right speech–motor related areas confirms previous evidence that right audio–speech areas are over-activated in *AWS*. Decreased beta2 *BC* in the right primary motor cortex is discussed in relation to abnormal neural mechanisms associated with time perception in *AWS*.

## Introduction

Stuttering is a developmental disorder of speech fluency that affects 1% of all adults (Craig et al., 2002). The behavioral manifestations of stuttering include unplanned sound prolongations, blocks in speech, and syllabic repetitions at the start of words and sentences. Stuttering is also associated with abnormalities in complex cognitive functions such as language (Weber-Fox and Hampton, 2008), motor preparation (Mersov et al., 2016), time perception (Ezrati-Vinacour and Levin, 2001), and also attention (Kamhi and McOsker, 1982).

Over the last two decades, a body of neuroimaging research has amassed which suggests that stuttering likely emerges from deficiencies in the brain mechanisms that support fluent speech production (e.g., Chang et al., 2009, 2017). Structurally, stuttering is related to several abnormalities in cortical and subcortical brain areas such as the Broca’s area (BA 44, 45), the basal ganglia, supplementary motor area, and parasylvian cortex (Gordon, 2002; Büchel and Sommer, 2004; Chang et al., 2009; Loucks et al., 2011; Sowman et al., 2017) which can be linked to mechanistic explanations proposed to account for stuttering such as auditory–speech dysfunction (Liotti et al., 2010; Jansson-Verkasalo et al., 2014) and also a speech–motor impairment (Neilson and Neilson, 1987). More recently, neural network-based investigations of stuttering have begun to provide a possible integrative account that might account for the large number of brain-based anomalies (for review see: Brown et al., 2005; Budde et al., 2014; Belyk et al., 2015; Etchell et al., 2017) now associated with stuttering.

Studies using connectivity analyses and graph theoretical methods have demonstrated network abnormalities in stuttering during resting state (Xuan et al., 2012; Ghaderi et al., 2016; Chang et al., 2017) that may involve anomalies in the default mode network (*DMN*) and affect attentional and executive functions (Chang et al., 2017). Such studies provide an insight into the neural correlates of psychological dysfunctions, particularly anxiety, that have long been associated with stuttering (Craig, 1990; Menzies et al., 1999; Iverach et al., 2011). Recent studies such as that by Yang and colleagues provide evidence that some of the hitherto unexplained neural abnormalities evident in stuttering might be attributable to the close association between stuttering and anxiety (Yang et al., 2017). Indeed, their results bear considerable concordance with other investigations that suggest emotional states and disorders (e.g., depression and anxiety) are associated with functional deficits in DMN activity (Coutinho et al., 2016; Messina et al., 2016).

To date, investigations of brain networks in stuttering have largely relied on functional magnetic resonance imaging (*fMRI*; Luc et al., 2008; Chang et al., 2009; Loucks et al., 2011). *fMRI* remains the gold standard for defining the topographic nature of network dysfunction in stuttering due to its excellent spatial resolution; however, as it has been suggested that stuttering depends on abnormal timing of brief durations and deficits in rapid movement control, planning, and preparation (e.g., Etchell et al., 2014, 2015; Wieland et al., 2015), neurophysiological methods that provide higher temporal resolution than *fMRI* may provide complementary information about the nature of neural connectivity in stuttering. Electroencephalography (*EEG*) can be used to acquire an ongoing record of the electrical activity of the brain with excellent temporal resolution, but since the origin of brain waves is a combination of post-synaptic potentials in the pyramidal cortical neurons, the source of *EEG* waves is not generally well reflected by the current distribution on the scalp. This means that inter-electrode connectivity analyses cannot easily be reconciled with *fMRI* network measurements (Babiloni et al., 2005; Mizuhara et al., 2005). Low-resolution brain electromagnetic tomography (*LORETA*) is an approach to solve inverse electromagnetic problem which transforms the *EEG* scalp current topography into a gross approximation of the *EEG* sources in brain space (Pascual-Marqui et al., 1994, 2011; Pascual-Marqui, 2002). Using *LORETA*, one can obtain a highly temporally-resolved neural signal that is mapped onto brain space.

Graph theoretical analysis (*GTA*) has become an important method for the study of complex systems in the field of neuroscience as well as in physics, astronomy, genetics, and engineering (Boccaletti et al., 2006; Bullmore and Sporns, 2009). *GTA* has been used to reveal the topological properties of structural brain networks and functional associations between brain regions. Important properties of neural information propagation and processing such as segregation and integration, and modularity and efficiency have been characterized by *GTA*. Graph theoretical indices such as the clustering coefficient, global efficiency, and small-worldness are meaningful neurobiological measures that can be calculated quickly (Rubinov and Sporns, 2010).

Small-world topography (Watts and Strogatz, 1998) provides an optimal balance between segregation and integration (Rubinov and Sporns, 2010). In 1998, Watts and Strogatz introduced the concept of small-world graphs based on Stanley Milgram’s works in the late 1960s (Watts and Strogatz, 1998; Boccaletti et al., 2006). Small-world graphs are simultaneously highly integrated and also highly segregated. Dynamically, these graphs exist in a specific state between random and regular graphs (Watts and Strogatz, 1998). Studies indicate that the functional and structural topography of the human brain, as well as other self-organizing systems, exhibit small-world properties (Watts and Strogatz, 1998; Achard et al., 2006; Bassett and Bullmore, 2006; Wang J. et al., 2009), the conformations of which are affected by developmental disorders (Wang L. et al., 2009; Barttfeld et al., 2011).

A more recently developed approach, minimum spanning tree (*MST*) analysis, is a powerful technique that can clarify emergent properties of functional brain networks (Stam, 2014). In weighted graphs there are many loops, which consist of sets of edges that connect a node to itself. The presence of loops in a graph increases the connectivity cost (the number of routes and edges between nodes). Spanning trees are subsets of graphs that cover all nodes without any loop (Stam, 2014; Tewarie et al., 2015). The *MST* is the tree with the lowest total cost (Graham and Hell, 1985); the unique, unweighted, binary graph that is made by the shortest path between all pairs of nodes without the occurrence of a loop. Therefore, the minimum connectivity cost involved in spanning all nodes is recovered by *MST* analysis (Tewarie et al., 2015). *MST* has been widely applied in the investigation of functional brain connectivity during tasks and rest (Stam et al., 2014; van Lutterveld et al., 2017).

This study represents the first attempt to use quantitative *EEG* (*QEEG*), *LORETA*, and graph theory in the field of language-related disorders. Graph theoretical analysis is applied here to investigate the brain’s topological network properties in adults who stutter (*AWS*). Further, we investigate possible relationships between the centrality of candidate brain regions and stuttering. *MST* analysis is used as the GTA approach. Based on previous reports that suggest that oscillatory brain activity in the beta band is abnormal in stuttering (Salmelin et al., 2000; Etchell et al., 2016; Mersov et al., 2016), we hypothesize that networks connected by coherent beta oscillations in stuttering will be compromised compared to those in controls. Since abnormal attention and executive network abnormalities have been observed in *AWS* (Chang et al., 2017) we also predict that language network abnormalities will be evident in *AWS* alongside abnormalities in the default mode and executive networks. This hypothesis is in line with previous findings that suggest stuttering is a disorder related to impaired working memory (Kaganovich et al., 2010), attention (Kaganovich et al., 2010), self-control (Eggers et al., 2013), and linguistic processing speed (Anderson and Wagovich, 2010).

## Materials and Methods

### Participants

Nineteen *AWS* [74% male, aged between 19 and 31 years, mean age (*SD*) 24.02 (3.65) years] and 52 age-, and gender-matched normally fluent speakers [*fluent*; 71% male, aged between 19 and 32 years mean age (*SD*) 24.47 (4.78) years] participated in this study. *AWS* participants were recruited from the Aftab Clinic in the city of Isfahan. Fluent speakers (controls) were recruited via an online announcement. All participants were native Persian speakers with no reported history of psychiatric/neurological disorders/diseases or use of medications that might affect neural function (e.g., medication for depression or seizure). All participants had normal hearing and normal or corrected-to-normal vision. At the time of testing, the Stuttering Severity Instrument for Adults—Fourth Edition (SSI–4) was administered by a speech therapist to each of the *AWS* and their stuttering severities were rated to be between mild and severe (Riley, 1972). A consent form was signed by all participants after the aim and procedure of the study was explained to them. The study conformed to the Helsinki declaration obligations and was approved by central ethical committee at Islamic Azad University.

### *EEG* Recording, Technical Setup, and Signal Pre-processing

*EEG* was recorded from 19 scalp electrodes (Fp1, Fp2, F3, F4, C3, C4, P3, P4, O1, O2, F7, F8, T3, T4, T5, T6, Fz, Cz, and Oz) positioned according to the 10–20 standard systems. A further two electrodes were positioned at left and right preauricular points (A1 and A2). *EEGs* were recorded using a Brain Master Discovery 24 amplifier and Electro-cap (eci). A linked-ear reference, commonly used in *QEEG* studies (Rotondi et al., 2016) was used. EEG cancelation is minimized by this montage (Sanei and Chambers, 2013). Impedance was kept under 5 kΩ during recording. Recordings were performed in an electromagnetically shielded faraday cage. *EEG* was digitized at 250 Hz and a low pass filter (40 Hz cutoff) was applied to avoid aliasing effects. Eight minutes of eyes open resting state *EEG* was recorded from each participant (Wu et al., 2010). Participants were instructed to avoid body or eye movements during the recording.

After recording, signals were screened for artifacts visually by an expert and then submitted to a z-score based artifact rejection algorithm implemented in the NeuroGuide software^1^. Twenty-five artifact-free segments (each segment was between 4 and 5 s in duration) were selected and exported for *LORETA* source localization.

### *LORETA* Analysis

Functional connectivity between 84 regions of interests (ROIs) was obtained by *LORETA* software version 20170220^2^. *LORETA* estimates cortical sources of brain waves based on the distribution of scalp-recorded potentials (Pascual-Marqui, 2002). This algorithm works as an inverse solution method and use a smoothness matrix that optimizes the solution (see Pascual-Marqui et al., 1994 for details). Recent versions of *LORETA* provide a connectivity utility (Pascual-Marqui et al., 2011). Functional dynamic connectivity of cortical regions with high temporal resolution can be calculated by *LORETA*. Although the number of *EEG* electrodes has a relationship to the precision of source estimation, a number of previous studies indicate that a reliable *LORETA* estimation can be achieved with only 19 channels (e.g., Thatcher et al., 2014; Aoki et al., 2015; Emory et al., 2015; Alahmadi et al., 2016; Clemens et al., 2016; Hata et al., 2016; Mohan et al., 2016b; Mumtaz et al., 2017). Time-resolved activity in all Brodmann areas excepting areas 12, 14, 15, 16, and 26 (localization of these regions is not implemented in the *LORETA* software) was estimated and the lagged coherences between 84 ROIs (42 Brodmann areas in the left hemisphere and 42 Brodmann areas in the right side) were computed for four frequency bands [theta (4–8 Hz), alpha (8–12 Hz), beta1 (12–20 Hz), and beta2 (20–30 Hz)]. The length of selected segments was at least 4 s and the sampling rate was 250 Hz (at least 1000 samples for each segment). The number of time frames per epoch selected in the *LORETA* software was 1024.

### Connectivity Measure and Adjacency Matrix

Non-instantaneous or lagged coherence is a methodological approach to frequency domain connectivity that removes the effects of volume conduction in *EEG* co-spectra (Pascual-Marqui, 2007; Pascual-Marqui et al., 2011). Lagged coherence has been used to investigate functional connectivity in resting-state brain networks in several disorders, e.g., Olbrich et al. (2013), Mohan et al. (2016a), Schwartz et al. (2016). However, to our knowledge, brain connectivity in *AWS* has not yet been investigated with lagged coherence.

**FIGURE 1:**
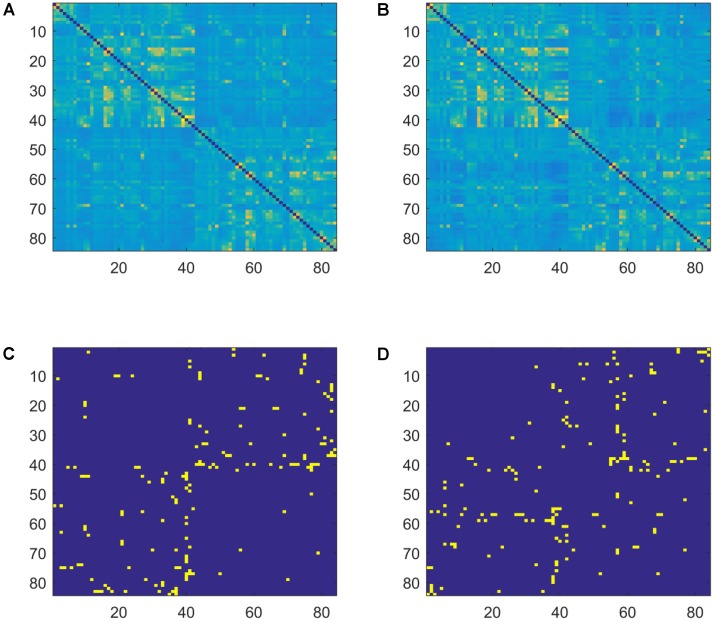
Weighted and sparse (*MST*) adjacency matrices for the two groups (*AWS* and control) in the beta2 band. **(A)** Averaged weighted adjacency matrix for controls. **(B)** Averaged weighted adjacency matrix for *AWS*. **(C)** Average *MST* adjacency matrix for controls. **(D)** Average *MST* adjacency matrix for *AWS*. Different patterns are visually observable between the *MST* graphs.

The 84 by 84 adjacency matrices were calculated separately in the theta, alpha, beta1, and beta2 bands. In the adjacency matrix, each row and each column represents a Brodmann area, and the lagged coherences between pairs of Brodmann areas are quantified at their intersections. Weighted and undirected adjacency matrices for the two groups (*AWS* and controls) are presented in **Figure 1**.

### Small-World Properties, Shortest Path, and Clustering Coefficient of Weighted Graph

Before 1998, graphs were classified generally as either random or regular (Boccaletti et al., 2006). In a random graph, edges are connected randomly to nodes and a node’s degree (the number of edges connected to each node) conforms to a Gaussian distribution (Boccaletti et al., 2006). Since the shortest path between nodes is typically small, random graphs are highly integrated. However, at the local level, there are no significant clusters between nodes and therefore random graphs are not segregated. Conversely, in a regular graph, all nodes have the same degree. In these graphs, the shortest path is long, and therefore, integration is minimal and segregation high.

In the majority of previous studies utilizing *GTA* analysis, small-world topography has been calculated for binary, non-weighted, and undirected graphs. This represents the simplest form of graph that requires the least computation and programing for calculation of small-worldness (Boccaletti et al., 2006; Humphries and Gurney, 2008). However, in a binary graph, there is only information pertaining to the existence or not of connections; connectivity strength between nodes is not visible.

Commonly, an adjacency brain connectivity matrix contains connectivity measures between nodes (electrodes or brain regions) that are not binary (e. g. coherence is a scalar that lies in the range 0 to 1; phase lag falls between -1 and +1, etc.). Therefore, the original adjacency brain connectivity matrix is a weighted matrix. For simplification, thresholding can be applied to transform weighted matrices to binary forms (Rubinov and Sporns, 2010; Mohan et al., 2016b; Ghaderi et al., 2017). In this approach, all of the matrix arrays with values higher than the threshold are replaced by 1 and all other array values set to zero. Although this approach has been used widely, e.g., Achard et al. (2006), Mohan et al. (2016b), Ghaderi et al. (2017), two basic problems are inherent in this approach. First, there is no specific threshold that must be used to make binary matrices, and therefore the choice of threshold can greatly influence the resulting structure of the graph (Tewarie et al., 2015). By systematically investigating the threshold “space,” any bias may be countered, but a second problem then arises in the statistical analysis where the use of multiple thresholds greatly increases the number of comparisons being made. To avoid these problems, analysis of weighted graphs has been proposed. Recently, a new measure, Small-World Propensity (*SWP*), has been proposed for identification of small-world properties of weighted networks (Muldoon et al., 2016). *SWP* compares clustering coefficient and characteristic shortest paths of a given network using lattice and random graphs:

$$\phi \;=\;1-\frac{\sqrt{\Delta_{C}^{2}+\Delta_{L}^{2}}}{2}$$

Where, Δ_C_is *C*_lattice_-*C*_given_ divided by C_lattice_-C_random_ and Δ_L_is *L*_given_-*L*_random_ divided by*L*_lattice_-*L*_random_. In this equation, Lattice and random graphs have the same size (number of nodes) and the same distribution of degree (probability distribution of degrees over all nodes) within the given network (Muldoon et al., 2016).

As indicated in the above equation, *SWP* is related to the characteristic shortest path (*L*) and clustering coefficient (*C*). *L* is the average minimum number of edges between all pairs of nodes. The clustering coefficient is defined by the number of triangles around a given node relative to the number of all neighbors (Boccaletti et al., 2006) and is closely related to brain modularity and segregation (Bullmore and Sporns, 2009; Rubinov and Sporns, 2010).

Here, random graphs with the same degree distribution and same size were made by permutation of adjacency matrices. Then *L*, *C*, and *SWP* were calculated for the stuttering group and control using a MATLAB toolbox at http://www.seas.upenn.edu/~dsb/ developed by Muldoon et al. (2016). For assessing *SWP* measures for the two groups, the *SWP* of 50 randomly permuted graphs was calculated and compared to the adjacency matrices.

### Minimum Spanning Tree (*MST*) and Integration Measures

The aforementioned problems involved thresholding the connectivity matrix are overcome by transforming the original weighted matrix into a unique sparse matrix. Functional brain connectivity using the *MST* approach can be quantified by the derived measures of maximum *BC*, nodal degree, leaf fraction (*LF*), diameter, and eccentricity (van Lutterveld et al., 2017).

*BC* is calculated by the counting all of the shortest paths that pass through a given node. Nodal degree is comparable to *BC* (it is simpler than *BC*). Degree is equal to the number of edges that are connected to a node. Nodes with a higher degree or *BC* play an important role in information processing within a graph (Boccaletti et al., 2006). It is suggested that a graph with higher maximum *BC* is more integrated (Bullmore and Sporns, 2009; Stam et al., 2014). LF relates to the integrative properties of the brain network (Stam et al., 2014; van Lutterveld et al., 2017). In a *MST*, the LF is equal to the number of nodes with degree 1 divided by *N* - 1, where *N* is the number of nodes in the graph. Therefore, a tree with a central node connected to all other nodes has maximum *LF* (equal to 1) and is highly integrated. Conversely, a tree with a series of one-to-one connected nodes exhibits the lowest possible *LF* (near zero) and also minimal integration (Stam et al., 2014). On the other hand, the maximum path length in a tree is defined as its diameter. Higher diameter is negatively associated with brain integration (Bullmore and Sporns, 2009; Stam, 2014). Eccentricity of a node is related to nodal isolation and average nodal eccentricity shows the tendency of nodes within the network to be isolated and poorly integrated (Rubinov and Sporns, 2010; Stam et al., 2014; van Lutterveld et al., 2017). Here, *MST* analysis on the 84 by 84 adjacency matrices was performed using MATLAB R2016a and the biograph toolbox. The measures of tree (e.g., *BC*, degree, *LF*, diameter, and eccentricity) were obtained via the brain connectivity toolbox developed by Rubinov and Sporns (2010)^3^.

### Statistical Analysis

Non-parametric permutation tests (Maris and Oostenveld, 2007) were applied to compare the between-subject measures of graph indices within frequency bands. Each permutation contained 5000 random shuffles. Seven graph indices (*SWP*, *L*, *C*, maximum *BC*, *LF*, diameter, and average eccentricity) were compared in four frequency bands (theta, alpha, beta1, and beta2) and then 28 (7 indices × 4 frequencies) independent permutation tests were performed. To minimize the possibility of false positives resulting from multiple comparisons, False Discovery Rate (*FDR*) correction was applied. The resulting *q*-values (corrected *p*-values in *FDR*) less than 0.05 were accepted as indicating statistically significant differences.

To evaluate the local corporation of cortical regions, *BC* and eccentricity of all Brodmann areas was investigated using a separate non-parametric permutation test. This latter analysis was performed only at the frequencies that maximum *BC* or/and average eccentricity was significant. *FDR* correction was also applied to minimize the likelihood of type I errors that might arise through comparison of 84 Brodmann areas over multiple frequencies. Non-parametric statistical tests were performed in MATLAB R2016a.

## Results

**FIGURE 2:**
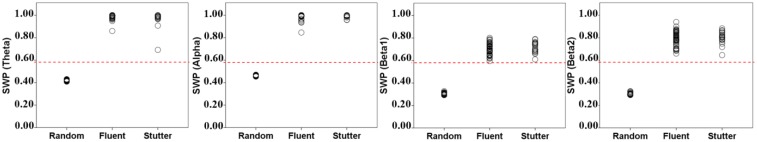
*SWP* measures for all samples in permuted random graphs (group 1), the control group (group 2), and the *AWS* group (group 3). The control and *AWS* groups exhibit values higher than 0.6, indicating that they have small-world connectivity. Permuted random graphs exhibit values less than 0.6.

**Figure 2** shows that both the controls and *AWS* exhibit *SWP* values over 0.6 in all frequency bands. Conversely, randomly permuted graphs show *SWP* less than 0.6. As suggested by Muldoon et al. (2016), *SWP* values over 0.6 are indicative of small-world networks. Therefore, all of the brain-based graphs (in both groups) exhibit small-world properties that are significantly different from random graphs (**Figure 2**).

After *FDR* correction, three graph indices exhibited significant differences between groups in the theta band and one measure was significantly different between groups in the beta2 band.

In the theta band a significant difference between groups occurred in the diameter of *MST* (*t* = -2.83, *P* = 0.0001). In this band, the *AWS* group had a smaller diameter (mean = 13.894, *SD* = 2.051) than the fluent control group (mean = 15.750, *SD* = 2.573). Significant differences were also observed between the two groups in the measures of characteristic shortest path (*t* = 2.71, *q* = 0.003) and clustering coefficient (*t* = -2.81, *q* = 0.002). The fluent group had a lower characteristic shortest path and a higher clustering coefficient (respectively: mean = 1.061, *SD* = 0.026; mean = 0.937, *SD* = 0.019) than the *AWS* group (respectively: mean = 1.080, *SD* = 0.029; mean = 0.922, *SD* = 0.023).

In the beta2 band, a significant difference was seen in the maximum *BC* of *MST* (*t* = 3.04, *q* = 0.007). The *AWS* group (mean = 5023.684, *SD* = 574.688) had a higher maximum *BC* than did the control group (mean = 4659.608, *SD* = 398.141). There were no significant differences in any measures in the alpha and beta1 bands between groups.

**FIGURE 3:**
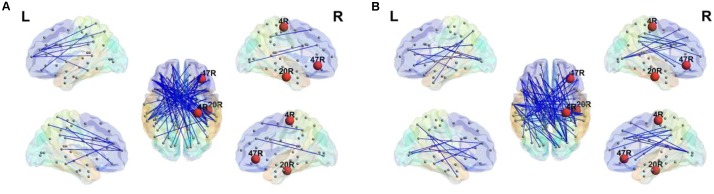
Minimum spanning tree in the beta2 band. Significant differences of *BC* (red nodes) were observed for right primary motor cortex (4R), right inferior temporal lobe (20R) and right inferior frontal gyrus (47R). The stuttering group **(A)** shows higher *BC* than the fluent group **(B)** in 20R and right 47R. The *AWS* exhibit lower *BC* in 4R. The figure was generated using BrainNet viewer toolbox version 1.53 (Xia et al., 2013).

Since there was a significant difference between groups in the maximum *BC* in the beta2 band, *BC* of *MST* was investigated for the 84 Brodmann areas. After *FDR* multiple comparison correction, significant differences between the stuttering group and controls were observed in the right hemisphere; primary motor cortex (BA4; *t* = -2.03, *q* = 0.001), inferior temporal lobe (BA 20; *t* = 3.02, *q* = 0.0001) as a part of the *DMN*, and a part of the inferior frontal gyrus (BA 47; *F* = 2.84, *q* = 0.0001). There was no significant difference in the *BC* in the left hemisphere. The normally fluent group exhibited higher *BC* in BA 4 than the *AWS* group. However, they showed lower values of *BC* in BA 20 and 47 than the *AWS* group. Significant differences in *BC* are visually presented in the **Figure 3**.

Weighted and sparse *MST* graphs for the beta2 band are presented in **Figure 1**. While the weighted graphs are visually similar between groups, differences are evident in the sparse *MST* matrices. Topological *MSTs* are presented in **Figure 3**.

## Discussion

These results indicate that alterations in very fast fluctuations and synchronization of post synaptic dipole arrangements, in various brain regions involved in generating *EEG* coherence, are associated with stuttering. Both groups studied here show small-world networks in the functional brain connectivity, and all participants exhibit *SWP* higher than 0.6. Since a graph with small-world topology exhibits an optimized and enhanced signal-propagation speed and synchronizability (Watts and Strogatz, 1998), optimized information transformation and propagation occurs in adult who stutter (*AWS)* as well as in controls. However, functional deficits in weighted graphs and *MST* analysis were observed in the theta and beta2 bands in *AWS*. Significant differences between controls and *AWS* in *BC* are also observable in the right primary motor area, inferior temporal lobe, and inferior frontal cortex. *AWS* show higher *BC* than controls in right temporal and inferior frontal areas and lower *BC* in right primary motor cortex. We discuss these results in the following two sections with regard to the functional meaning of brain oscillations and the regions involved in the observed functional connectivity deficits in *AWS*.

### Alpha Wave: The Role of Emotion in Stuttering

The role of alpha activity in emotional states such as anxiety (Boutcher and Landers, 1988; Knyazev et al., 2006) and depression (Gotlib, 1998; Fingelkurts et al., 2007) has been widely investigated. Several studies suggest that those who stutter exhibit higher levels of anxiety (e.g., social anxiety or social phobia) than people whose speech is fluent (Mahr and Torosiana, 1999; Messenger et al., 2004; Iverach and Rapee, 2014). However, our results indicate that, at least during resting conditions, *AWS* exhibit alpha connectivity that is not different from that seen in control subjects. This result is consistent with previous studies that explain anxiety in stuttering as a secondary reaction (Alm, 2004). In the context of a possible alpha difference in *AWS* that might be based on reactive anxiety, then, in the current study where the *AWS* were at rest, it might not be expected that alpha network differences between groups would be evident.

### Theta Wave: The Role of Executive Network in Stuttering

In the theta band, *AWS* have a higher value of characteristic shortest path and a lower value of clustering coefficient than controls, suggesting that the theta network is disrupted at both local and global levels in *AWS*. Theta-mediated networks in *AWS* are less integrated and also less segregated.

Theta activity is closely associated with executive functions such as problem solving, planning, working memory, and also attention (Sauseng et al., 2005; Mizuhara and Yamaguchi, 2007). Further, functional connectivity in the theta band is related to activity in central executive networks (Sauseng et al., 2004, 2005) and abnormal theta connectivity during rest state may suggest impaired *DMN* function (Scheeringa et al., 2008). Abnormal functional connectivity in the theta network is reported in attention-related disorders such as attention deficit hyper activity disorder (*ADHD*) during rest (Ghaderi et al., 2017) and during tasks (Sauseng et al., 2007). Similar to our current findings in *AWS*, in *ADHD* impaired functional segregation and integration in the theta network is evident (Ghaderi et al., 2017). Such similarities reinforce the hypothesis that there is a neural basis for abnormal executive functions among the stuttering group that may explain previous findings that indicate impairment of working memory (Kaganovich et al., 2010), attention (Kaganovich et al., 2010), self-control (Eggers et al., 2013), and also linguistic processing speed (Anderson and Wagovich, 2010) in stuttering. This result may also help explain the reported treatment effects of attentional training (Nejati et al., 2013) and also mindfulness (Boyle, 2011) in stuttering.

*MST* analysis indicates that there is no regional theta-mediated abnormality in *AWS*. This finding, in combination with the aforementioned local and global anomalies of the theta network, may suggest that attentional deficits in *AWS* are related to the functional connectivity of the whole brain rather than those within a specific module (e.g., middle frontal lobe as a hub in executive network).

### Beta Wave: The Role of Motor Timing and Audio–Speech Regions

Many studies have investigated the role of motor, speech and auditory related impairments in stuttering, e.g., (Büchel and Sommer, 2004; Watkins et al., 2008; Chang et al., 2009). These confirm motor–speech (Watkins et al., 2008) and audio–speech (Luc et al., 2008; Chang et al., 2009) deficits exist, at least at the neural level, in stuttering. The current results reaffirm that functional brain differences in *AWS* occur in both primary motor related regions and also audio-speech areas. We show that the *BC* of beta2 mediated connections is decreased in the right primary motor cortex in *AWS*. A node with high *BC* lies on a large number of shortest paths. Nodes with significant association in information transfer often have high *BC* while *BC* is zero for a dead-end node (Barthelemy, 2004; Rubinov and Sporns, 2010). Decreased *BC* in right primary motor cortex suggest that this area plays a reduced role in neural communication and information propagation within the cortico-cortical networks in *AWS*. This result is comparable with previous findings that show hypo-activity of the cortical motor and premotor areas in stuttering (Salmelin et al., 2000; Watkins et al., 2008). Previous findings suggest that abnormal motor and pre-motor activity during speech tasks may be causal in stuttering (Packman, 2012). However, the present results were obtained during resting state and could therefore fit with an explanation that posits *DMN* deficits in stuttering (Xuan et al., 2012; Chang et al., 2017).

Recently it has been suggested by Etchell et al. (2014, 2016) that stuttering is a timing deficit disorder underpinned by abnormal functioning of beta-mediated timing networks (see also Alm, 2004). According to the broader literature in this area (Buhusi and Meck, 2005; Fujioka et al., 2009; Kononowicz and van Rijn, 2015; Merchant and Bartolo, 2017), beta activity in the basal ganglia-thalamocortical circuits (measured by *EEG/MEG* from central brain locations) can be considered in relation to interval timing. Behaviorally, deficits in response timing tasks are frequently reported in people who stutter (Ezrati-Vinacour and Levin, 2001; Olander et al., 2010; Falk et al., 2015) and recently it has been suggested that impairment of resting state functional connectivity is involved in time discrimination deficits in stuttering (Chang et al., 2016). Since beta activity in primary and supplementary motor cortices is associated with activity of basal ganglia-thalamocortical circuit (Kropotov, 2010), decreased *BC* of primary motor cortex in resting state beta2 adds further, albeit indirect, neural evidence for a deficit in interval timing networks in stuttering that involve the striatum.

*AWS* exhibit increased beta2 *BC* in the right speech–motor related areas during resting state. This result suggests that extra information is transferred via right audio–speech regions and that hyper propagation of beta signals occurs in this region. Increased activity of right audio–speech areas has previously been observed in studies using *fMRI* (Luc et al., 2008; Chang et al., 2009). Therefore, as suggested by other authors (Luc et al., 2008; Chang et al., 2009), AWS may require more neural activity in right audio–speech regions to compensate for deficiencies elsewhere. It could be posited that decreased signal propagation in the right primary cortex causes an imbalance in the neural network and hence shortest paths are transferred from right audio–speech regions.

### Study Limitations

Two limitations that affect this study should be considered. Firstly, as LORETA accuracy is dependent to an extent on the EEG montage density, the relatively small number of electrodes we were limited to suggest that some caution regarding interpreting absolute source localization accuracy should be exercised. However, this is always the case with EEG source analysis, and the fact that the results presented here are both physiologically plausible and in strong concordance with previous studies mitigate this concern. Furthermore, whilst there is some evidence that suggests montage density positively correlates with deep source reconstruction accuracy, a clear relationship to reconstruction of superficial sources is less clear (Liu et al., 2018). Secondly, whilst not an absolute limitation, our choice of referencing scheme, the linked ear montage, whilst widely used in similar studies e.g., Hata et al. (2016), is not universally accepted as the best option for EEG studies in source space. Future studies would be advised to systematically address the implications of the choice of reference scheme on possible functional connectivity deficits in AWS.

## Conclusion

Our results reinforce previous findings that DMN deficits occur in stuttering (Xuan et al., 2012; Chang et al., 2017). Altered networks found in *AWS* include attentional circuits, primary motor regions and also audio–speech related areas. We found decreased functional integration and segregation comparable to that seen in other developmental disorders (Ghaderi et al., 2017) but no local impairments in specific regions were evident. *AWS* also show impairment in the beta network in primary motor cortex and audio-speech areas. We suggest that abnormal activity in the beta network may relate to timing deficits and hypo-activation of motor related areas.

## Author Contributions

AG performed the main analysis, data recording, and wrote the main body of the manuscript. MA performed a part of graph analysis and he collaborated in data recording. PS managed the methodology, edited the manuscript, and also wrote a considerable part of the Section “Discussion.”

## Conflict of Interest Statement

The authors declare that the research was conducted in the absence of any commercial or financial relationships that could be construed as a potential conflict of interest.

## Footnotes

1 http://www.appliedneuroscience.com

2 http://www.uzh.ch/keyinst/loreta.htm

3 http://www.brain-connectivity-toolbox.net
